# COVID-19 epidemic and public health interventions in Shanghai, China: Statistical analysis of transmission, correlation and conversion

**DOI:** 10.3389/fpubh.2022.1076248

**Published:** 2023-01-10

**Authors:** Dali Yi, Xicheng Chen, Haojia Wang, Qiuyue Song, Ling Zhang, Pengpeng Li, Wei Ye, Jia Chen, Fang Li, Dong Yi, Yazhou Wu

**Affiliations:** ^1^Department of Health Statistics, College of Preventive Medicine, Army Medical University, Chongqing, China; ^2^Department of Health Education, College of Preventive Medicine, Army Medical University, Chongqing, China

**Keywords:** COVID-19, public health, interventions, dynamic prevention and control, effective regeneration number

## Abstract

**Background:**

The Shanghai COVID-19 epidemic is an important example of a local outbreak and of the implementation of normalized prevention and disease control strategies. The precise impact of public health interventions on epidemic prevention and control is unknown.

**Methods:**

We collected information on COVID-19 patients reported in Shanghai, China, from January 30 to May 31, 2022. These newly added cases were classified as local confirmed cases, local asymptomatic infections, imported confirmed cases and imported asymptomatic infections. We used polynomial fitting correlation analysis and illustrated the time lag plot in the correlation analysis of local and imported cases. Analyzing the conversion of asymptomatic infections to confirmed cases, we proposed a new measure of the conversion rate (*C*_*r*_). In the evolution of epidemic transmission and the analysis of intervention effects, we calculated the effective reproduction number (*R*_*t*_). Additionally, we used simulated predictions of public health interventions in transmission, correlation, and conversion analyses.

**Results:**

(1) The overall level of *R*_*t*_ in the first three stages was higher than the epidemic threshold. After the implementation of public health intervention measures in the third stage, *R*_*t*_ decreased rapidly, and the overall *R*_*t*_ level in the last three stages was lower than the epidemic threshold. The longer the public health interventions were delayed, the more cases that were expected and the later the epidemic was expected to end. (2) In the correlation analysis, the outbreak in Shanghai was characterized by double peaks. (3) In the conversion analysis, when the incubation period was short (3 or 7 days), the conversion rate fluctuated smoothly and did not reflect the effect of the intervention. When the incubation period was extended (10 and 14 days), the conversion rate fluctuated in each period, being higher in the first five stages and lower in the sixth stage.

**Conclusion:**

Effective public health interventions helped slow the spread of COVID-19 in Shanghai, shorten the outbreak duration, and protect the healthcare system from stress. Our research can serve as a positive guideline for addressing infectious disease prevention and control in China and other countries and regions.

## Introduction

In December 2019, coronavirus disease 2019 (COVID-19), caused by severe acute respiratory syndrome coronavirus 2 (SARS-CoV-2), emerged in China and spread to all parts of the world (1–4). COVID-19 causes symptoms such as fever, cough, fatigue, shortness of breath, and pneumonia, which can lead to death in severe cases. COVID-19 had spread to the vast majority of countries by May 2022, with over 500 million confirmed cases and over 6 million deaths, having a profound impact on politics, economies, and societies around the world.

To effectively control the COVID-19 outbreak, China responded with a policy of “dynamic clearing and social clearing.” However, with the continuous variation in the virus and the complex situation of the international environment, a small-scale outbreak and rebound of the epidemic were inevitable (5, 6). In late February 2022, a new round of local COVID-19 infections occurred in Shanghai. Shanghai is China's most important economic center, and if the outbreak were to spread to other parts of the country, the consequences would be incalculable. On March 28, 2022, the Shanghai government gradually implemented public health intervention measures in the city to curb the spread of the epidemic, including closed district management, paying attention to elderly individuals, establishing designated hospitals and carrying out double-antibody screening (7, 8). In late May, the epidemic situation in Shanghai was essentially controlled, and normal production and life were restored on June 1. The epidemiological characteristics of the Shanghai epidemic and the effect of public health interventions are still unclear, and there are few relevant studies (9). Thus, the association cannot be comprehensively and accurately described.

The Shanghai epidemic was quite different from that in Wuhan (December 8, 2019, to March 8, 2020) (10). Reviewing the progress of the epidemic in Shanghai, inadequate control of imported cases from abroad was an important cause of the outbreak. As a result of the epidemic in Hong Kong (December 31, 2021, to March 23, 2022) (11), Shanghai had taken on the responsibility of transporting some imported personnel to Shenzhen. The epidemic was sparked by an increase in the number of imported personnel and flaws in isolation management. In addition, the proportion of asymptomatic infections in Shanghai was significantly higher than that in Wuhan. Therefore, it is necessary to analyze the correlation between local cases and imported cases as well as the conversion between confirmed cases and asymptomatic infections (12, 13).

Many studies have calculated the basic regeneration index (*R*_0_) of the COVID-19 epidemic, and its estimated value is generally in the range of 2–7, revealing the high infectiousness of COVID-19 (14–16). However, due to the small number of cases and regions, more research is needed to confirm this finding. The effective reproductive number (*R*_*t*_) refers to the average number of new cases that can be caused by one case at time *t*. It can reflect the epidemic trend of infectious diseases in real time, and it is an important index to guide epidemic prevention and control and to evaluate intervention measures (17–19). However, there is a lack of relevant research evaluating the developmental trend of *R*_*t*_ and the effect of public health intervention measures in Shanghai in 2022. We use statistical methods to investigate and quantify changes in the epidemiological characteristics of the spread of COVID-19 in Shanghai as well as the effects of public health interventions, with the goal of developing a comprehensive assessment system for the disease process, disease transmission, and the impact of control measures that will serve as a foundation for future interventions. Policy formulation provides a scientific foundation for accuracy and operability as well as significant promotional value.

Therefore, we use Shanghai, China, as a case study to examine the epidemiological characteristics and the impact of public health interventions against the backdrop of normalized prevention and control to provide a positive guideline for the follow-up response to the 2022 Shanghai epidemic. The main work and contributions of this paper are as follows.

(1) We conducted extensive research on the main period of the epidemic in Shanghai (January 30 to May 31, 2022), taking into account cluster analysis and public health interventions to divide the development stages of the epidemic, with the goal of analyzing the epidemic situation in Shanghai. Changes in transmission characteristics and control measures as well as their correlations were investigated. (2) Because the Shanghai epidemic may have been caused by imported cases and there were many asymptomatic infections, we examined not only the correlation between local and imported cases but also the conversion of confirmed cases and asymptomatic infections. The difference between the Shanghai epidemic analysis and previous epidemic analyses, as well as the innovation of this study compared to previous research, lies in the two types of analysis. (3) We used effective reproduction number (*R*_*t*_) analysis to determine the impact of public health interventions on epidemic prevention and control, with the goal of evaluating the temporal correlation between public health interventions and Shanghai epidemic prevention and control and then analyzing the significance and correctness of public health interventions. (4) We simulated and predicted the evolution of the epidemic when the implementation of interventions was delayed, and we examined the number of cases that could have been avoided due to public health interventions. This study confirms the timeliness and effectiveness of the interventions and provides a new model and experience related to the global fight against the Omicron-based epidemic.

## Materials and methods

### Data sources

The data and public health interventions in this study were public data released by the National Health Commission and the Shanghai Municipal Health Commission. Newly added cases were classified as local confirmed cases, local asymptomatic infections, imported confirmed cases and imported asymptomatic infections. In this outbreak, a local case was first found on March 1. Since this round of the epidemic came from abroad, we collected case data starting from January 30. On June 1, Shanghai announced the restoration of normal production and everyday life. Therefore, the data that we collected covered the period from January 30 to May 31, 2022, totaling 121 days.

### Statistical analysis

#### Transmission analysis

We used cluster analysis to aid segmentation and plotted dynamic time-series maps to improve the interpretability of the intervention phase. As feature vectors, we used the number of newly added cases of local confirmed cases, local asymptomatic infections, imported confirmed cases, and imported asymptomatic infections, and we grouped the samples using time as the label. The Manhattan distance method was used to create a hierarchical clustering of the closest dates. In the rectangular coordinate system, the date and the number of infected people are denoted by *X* and *Y*, respectively. Assuming that there are points *i* of coordinate (*X*_1_, *Y*_1_) and *j* of coordinate (*X*_1_, *Y*_2_) on the plane, the Manhattan distance *D*(*i, j*) between them is expressed as follows:


(1) $$\begin{array}{l} D\left(i,j\right)=|X_{1}-X_{2}|+|Y_{1}-Y_{2}| \end{array}$$

we used *R*_*t*_ analysis based on the Poisson distribution to determine the transmission capacity of each stage and the impact of public health interventions. *R*_*t*_ is defined as the average number of secondary cases of primary cases in the population at time *t*, representing the average number of second-generation cases that an infected person diagnosed at a certain time will infect during the infection period (20, 21). *R*_*t*_ can be used to measure the real-time transmissibility during an epidemic and to evaluate viral transmission before and after intervention measures. The *R*_*t*_ at the beginning can be defined as *R*_0_, and the *R*_*t*_ at the end can be defined as *R*_final_.

We used the EpiEstim package in R software (version 3.6.3) to fit *R*_*t*_ and the 95% confidence interval (CI) using the number of new cases reported daily. *R*_*t*_ can be expressed as follows:


(2) $$\begin{array}{l} R_{t}=\frac{I_{t}}{\sum_{s=1}^{t}I_{t-s}w_{s}} \end{array}$$

Here, *I*_*t*_ represents the number of new cases generated at time *t*; $\sum_{s=1}^{t}I_{t-s}w_{s}$ represents the sum of the infection incidence up to time (*t* – 1); and *w*_*s*_ represents the probability function of the serial interval (SI). The infectious characteristics of infected individuals are the basic idea of *R*_*t*_ calculation, and the specific principle is shown in references (22, 23). In short, *R*_*t*_ can be calculated by dividing the proportion of new cases at time *t* by the cumulative cases at time (*t –* 1), and its weight is*w*_*s*_. Assuming *R*_*t*_ has a gamma prior distribution, Bayesian statistical inference using the Poisson distribution can generate a posterior distribution of *R*_*t*_. The steps in the calculation can be summarized as follows: (1) Determine the SI of the epidemic situation, including the mean and standard deviation. (2) Determine the sliding window time length, and estimate the SI using the previously studied SI distribution. That is, the infection time interval in two consecutive generations follows the Gamma distribution with a mean value of 4.87 and a standard deviation of 0.65 when a 1-day moving window is used. (3) The plot function is used to plot the change in *R*_*t*_ over time.

#### Correlation analysis

In the time-series correlation analysis of domestic and imported cases, we used polynomial fitting to obtain the fitting curve and performed time lag analysis. Here, *x* and *y* represent the number of local and imported cases at a given time. The overall sample with *m* time points can be written as follows:


(3) $$\begin{array}{l} \left\{\left(x_{1},y_{1}\right)\left(x_{2},y_{2}\right)\cdots \left(x_{m},y_{m}\right)\right\} \end{array}$$

Here, a sample time point can be expressed as follows:


(4) $$\begin{array}{l} \left(x_{i},y_{i}\right),i=1,2,3,\ldots ,m \end{array}$$

When the distribution of these points resembles the graph structure of a polynomial of degree n, the final fitting formula is as follows:


(5) $$\begin{array}{l} ŷ=a_{0}x^{n}+a_{1}x^{n-1}+a_{2}x^{n-2}+\cdots +a_{n-1}x+a_{n} \end{array}$$

Here, *X* and *Y* represent the number of local and imported cases at a certain time and construct a polynomial fitting formula; *a*_0_ – *a*_*n*_ represent the fitting coefficient; *n* in *x*^*n*^ represents the index of the fitting polynomial; and ŷ represents the fitting value of the corresponding imported case when the local case is *x*.

We used interval geographic maps and nuclear density maps to describe the incidence distribution in each region and the time point in the spatial distribution. Kernel density estimation, which analyzes the density distribution of each element in the observed object's corresponding geospatial domain, was conducted based on ArcGIS 10.8.1.

#### Conversion analysis

Asymptomatic infections account for a relatively high proportion of the overall infections, which is not only an important feature of this epidemic but also an important basis for studying the impact of public health interventions. To better analyze the ratio of asymptomatic infections to confirmed cases and to analyze the changing characteristics of the epidemic as it developed, we innovatively propose the concept of the “conversion rate.” If the incubation period is defined as *t*, the number of converted cases (i.e., the number of confirmed cases) on that day is *N*_1_, and the number of untransformed cases (i.e., the number of asymptomatic infections) within *t* days before that day is *N*_2_. Thus, the conversion rate *C*_*r*_ can be defined as follows:


(6) $$\begin{array}{l} C_{\text{r}}=\frac{N_{1}}{N_{2}} \end{array}$$

Because the exact incubation period for transitioning from an asymptomatic infection to a confirmed case is currently unknown, we used a variety of settings, including 3, 7, 10, and 14 days, to better analyze the law of conversion. Next, we used simulation studies to validate the significance of public health interventions. Assuming that no public health intervention measures were implemented after March 28, the number of cases continued to rise in line with the *C*_*r*_ value (3.02%) on March 28, and the conversion number of asymptomatic infections can be calculated accordingly.

## Results

### Division of development stages

**Figure 1:**
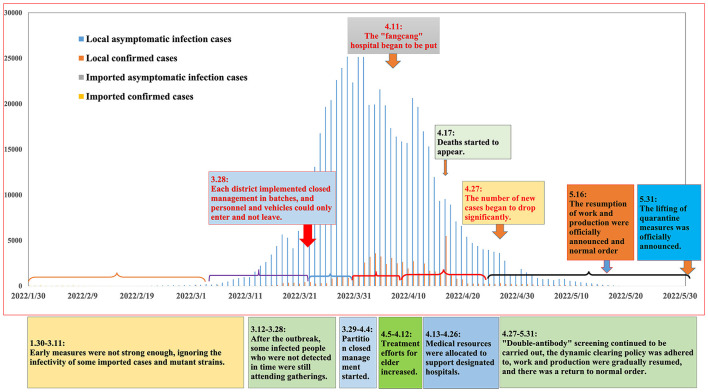
Correlation analysis between daily cases and public health intervention. Based on the date of symptoms, the epidemic curve shows the number of cases and events per day. We also further describe the key events, situation characteristics and details of public health interventions at each stage.

We conducted cluster analysis based on the total number of newly added cases per day. We comprehensively considered the optimal clustering results and the public health intervention time points and then divided the main epidemic period into six stages. Figure 1 illustrates the epidemic curve of the symptom occurrence date and key intervention events.

Regarding the first stage (1.30–3.11), sporadic infections began to appear and were mainly imported cases, and sporadic local cases began to appear. The imported cases were not controlled in a timely manner, and cryptic transmission likely began at this time, leading to the local transmission of COVID-19. Regarding the second stage (3.12–3.28), local asymptomatic cases and confirmed cases showed an upward trend. On March 24, thousands of new asymptomatic infections were reported every day for the first time.

Regarding the third stage (3.29–4.04), the epidemic rapidly worsened, and the government conducted nucleic acid screening and implemented personnel and traffic controls, further reducing the population's social mobility. The fourth stage (4.05–4.12) represented the high-risk period of the outbreak, with the number of newly local confirmed cases exceeding 1,000 per day and the number of asymptomatic infections exceeding 20,000 per day.

In the fifth stage (4.13–4.26), the epidemic was in the remission period. There was a delay in the effectiveness of public health intervention measures, and the incidence trend had been alleviated at this stage. The sixth stage (4.27–5.31) represented the epidemic control period. In local cases, newly confirmed cases and asymptomatic infections were gradually controlled to < 100 cases every day.

### Transmission analysis using the effective reproduction number

**Figure 2:**
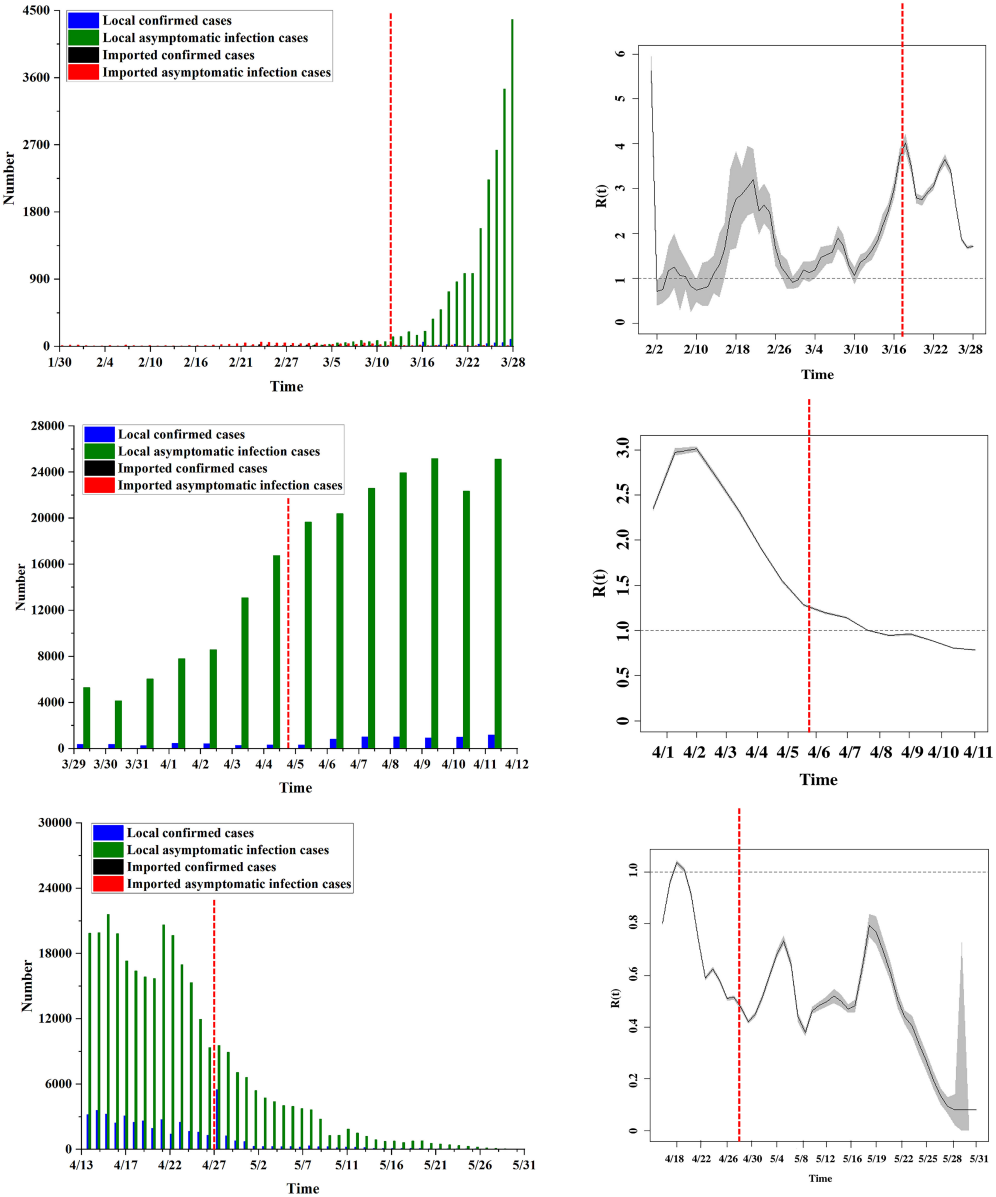
The shifting pattern of *R*_*t*_. In the six stages, the daily new cases and the change trend of the *R*_*t*_ value are compared.

We combined the new case map and the *R*_*t*_ change map (the sum of both local and imported cases) in the six stages to analyze the epidemic transmission characteristics in each stage, as shown in Figure 2.

In the first stage, *R*_*t*_ rose sharply and reached a maximum value on February 17 (*R*_*t*_ = 3.19). At this stage, *R*_*t*_ was mainly distributed between 1 and 3. In the second stage, *R*_*t*_ continued to rise and reached an overall peak on March 16 (*R*_*t*_ = 4.02). At this stage, *R*_*t*_ was mainly distributed between 2 and 4.

In the third stage, asymptomatic infections began to appear, causing fluctuations in *R*_*t*_. *R*_*t*_ showed a trend of first increasing and then decreasing. It reached a peak in this stage on April 1 (*R*_*t*_ = 3.01) and then gradually decreased, but *R*_*t*_ remained above 1.5. This stage is when the public health intervention measures began to take effect. In the fourth stage, *R*_*t*_ continued to decline, and it fell below 1 on April 9 (*R*_*t*_ = 0.99), indicating that the spread of the epidemic had reached a controllable range.

In the fifth stage, *R*_*t*_ rebounded in a small range of cases, but it was controlled 2 days later. *R*_*t*_ increased to above 1 on April 18 (*R*_*t*_ = 1.04) and decreased to below 1 on April 20 (*R*_*t*_ = 0.91). In the sixth stage, the fluctuation trend of *R*_*t*_ was obvious, but *R*_*t*_ was below 1.

In general, from February 28 to April 10, affected by imported cases from abroad, the average *R*_*t*_ in Shanghai was higher than the epidemic threshold, lasting ~5.5 weeks. Following the implementation of public health intervention measures in Shanghai (March 28), the estimated *R*_*t*_ generally decreased and fell below the epidemic threshold on April 10. The overall *R*_*t*_ level in the first three stages was higher than 1, and the overall level in the last three stages was lower than 1.

**Figure 3:**
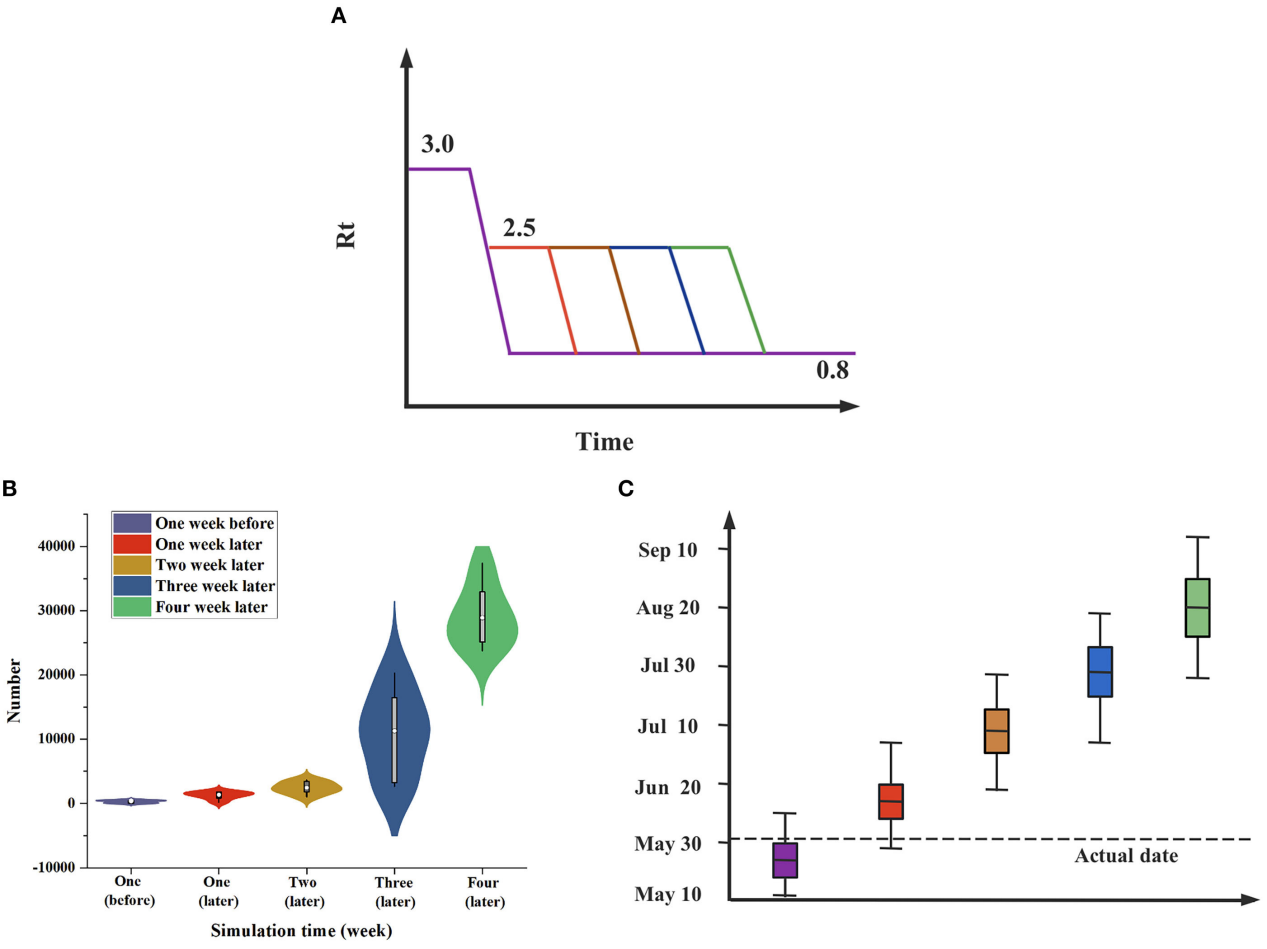
*R*_*t*_-based simulation prediction. **(A)** *R*_*t*_ is assumed to decline linearly over a 1-week period from *R*_0_ to *R*_final_. Estimates in advance simulations are obtained by considering *R*_0_ = 3.0 and *R*_final_ = 0.8; estimates in deferred simulations are obtained by considering *R*_0_ = 2.5 and *R*_final_ = 0.8. **(B)** Differences in the number of cases (when compared to actual values) when public health interventions were implemented 1 week earlier or 1–4 weeks later. **(C)** The case end date if public health interventions began 1 week earlier or 1–4 weeks later.

Furthermore, as shown in Figure 3, we examined the change in the number of predicted cases (compared to the actual value) and the end time of the epidemic under various simulation conditions. The predicted number of cases was reduced by 664 when the public health interventions were implemented 1 week earlier, and the epidemic was expected to end 9 days sooner. The number of predicted cases increased by 1,265, 2,450, 10,811, and 29,073 when the public health interventions were delayed by 1, 2, 3, and 4 weeks, respectively, and the epidemic was expected to end 18, 35, 56, and 63 days later, respectively.

### Time-series correlation analysis of imported and local cases

**Figure 4:**
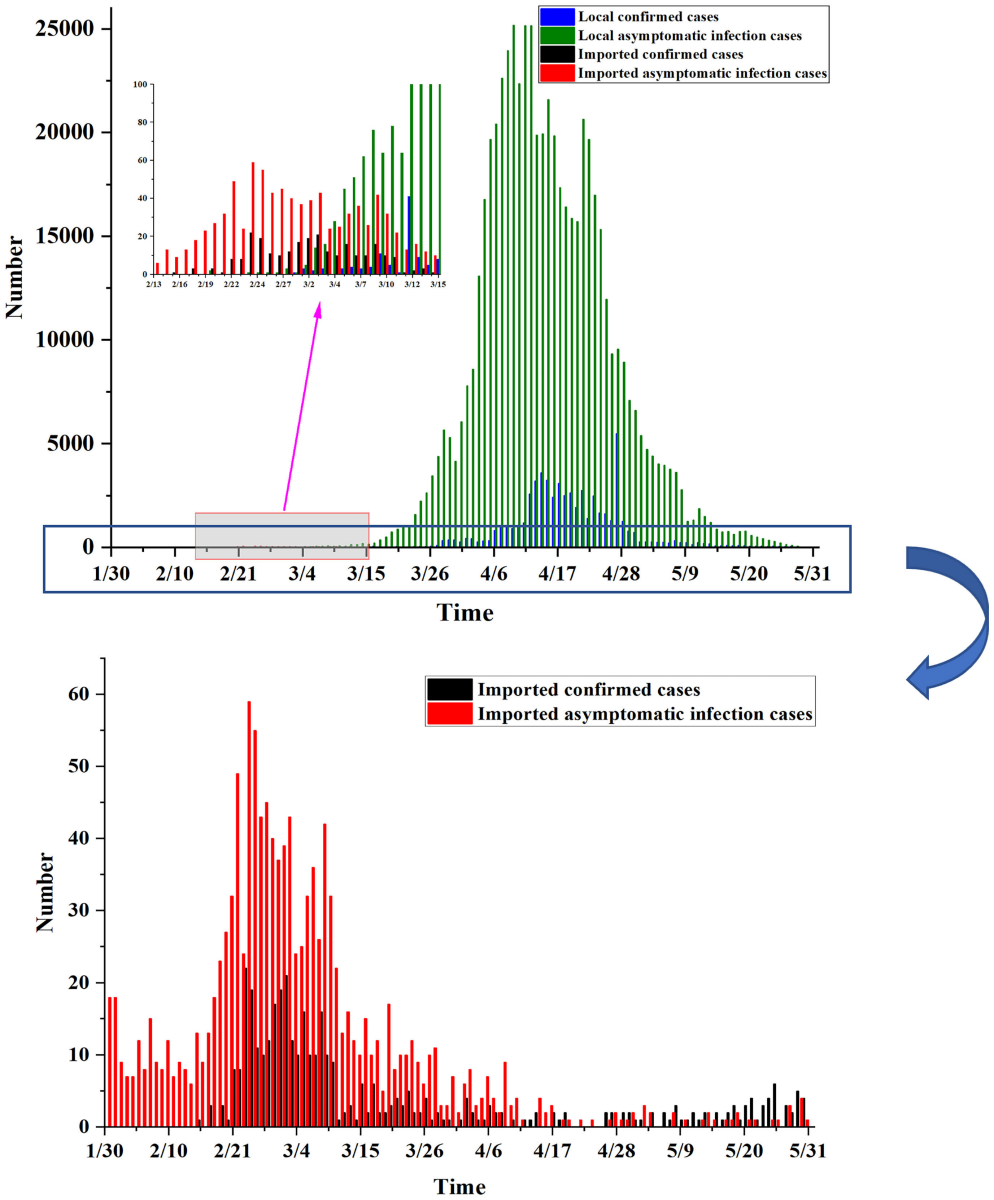
The temporal distribution of the epidemic. The time of onset and the epidemic curve of the Shanghai epidemic: Based on the daily number of new cases listed on the report date, the cases were divided into local confirmed cases, local asymptomatic infections, imported confirmed cases and imported asymptomatic infections.

We analyzed the time distribution characteristics of epidemic development in Shanghai, as shown in Figure 4. The epidemic curve shows the bimodal epidemic of local and imported cases. The earliest local confirmed cases occurred on March 1, and the largest number of cases (5,487) in a single day occurred on April 28. As Shanghai undertook the task of transporting imported cases, imported confirmed cases continued to exist, and more than 20 cases occurred in a single day on February 20, with the largest number of cases (59) on February 24. The 95% CIs of the four types of cases were calculated, and their main concentration dates were analyzed, as shown in Table 1. The results show that compared with imported cases, local cases had a certain time lag effect of approximately 1 month.

**Table 1:** Distribution analysis of daily new incidences of four types of cases.

**Type**	**Distribution date**	**Average value**	**95% CI**	

			**Lower bound**	**Upper bound**
Local confirmed cases	3.29~5.1	475.41	305.97	644.85
Local asymptomatic infection cases	3.27~5.8	4,847.13	3,496.70	6,197.57
Imported confirmed cases	2.5~4.3	10.42	8.03	12.81
Imported asymptomatic infection cases	2.18~3.25	3.25	2.39	4.10

**Figure 5:**
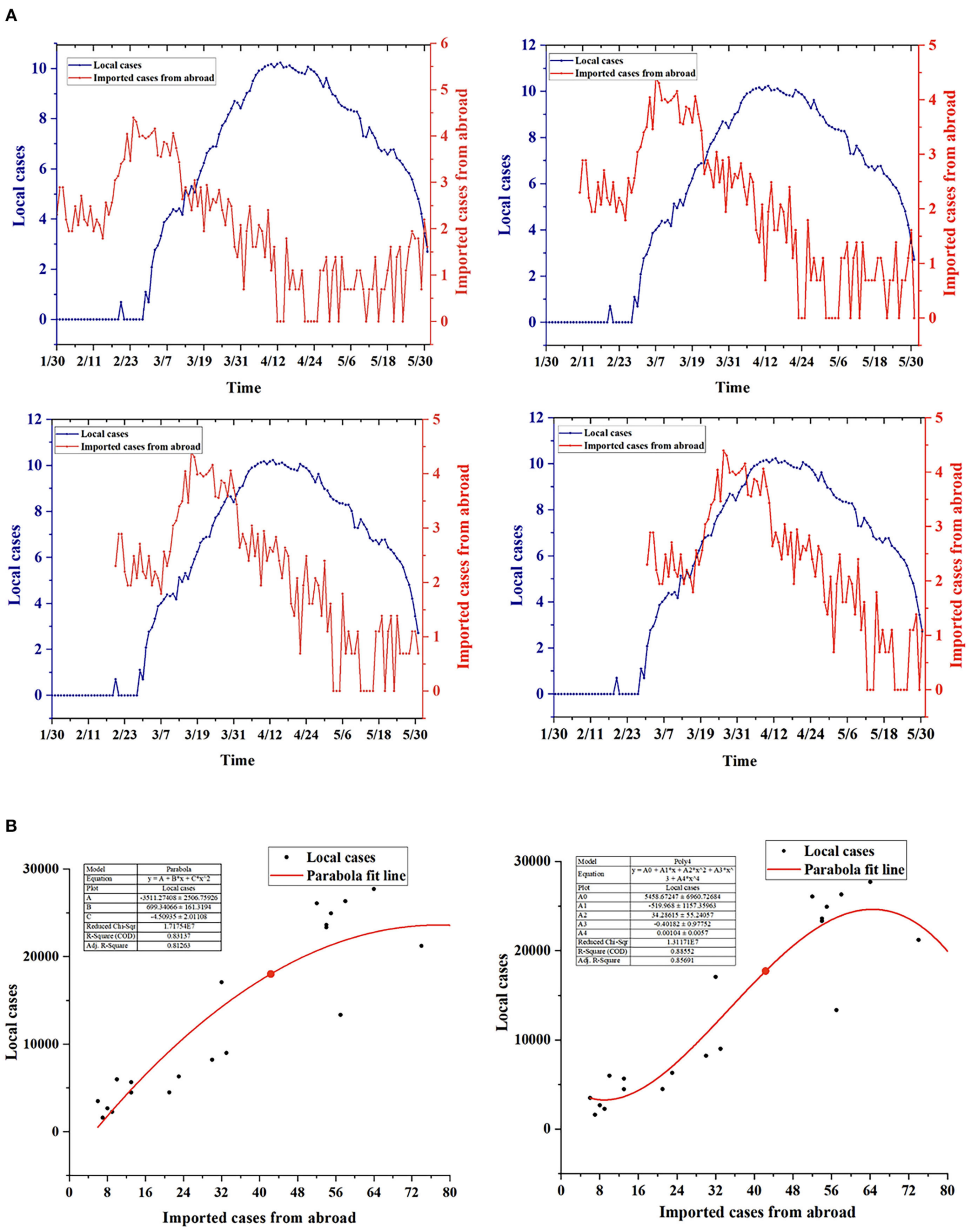
Correlation analysis between local cases and imported cases. **(A)** Time lag analysis of the change trend between local cases and imported cases: There was a large difference in the magnitude of factors. Natural logarithm processing was applied to local and imported cases. Based on the time of imported cases, the order was as follows: January 30, February 10, February 20 and March 2. **(B)** Correlation analysis between local cases and imported cases: The included local cases ranged from March 24 to April 13, and the imported cases ranged from February 11 to March 3.

According to the above results, there were mainly imported cases in the first stage. Shanghai had not strictly controlled and managed imported infected persons, and the number of imported cases was higher than that of local cases. During the second stage, local cases continued to rise. Local cases occurred with the emergence of imported cases, and there was a certain time lag effect of approximately 31 days. We analyzed the correlation between the number of imported and the number of local cases (including both confirmed cases and asymptomatic infections) and drew time lag analysis charts and correlation analysis charts, as shown in Figure 5. In the time lag analysis chart, compared with imported cases, local cases had a greater lag and amplification, and the lag period was approximately 31 days, confirming the previous speculation. In the correlation analysis chart, the curves fitted by various methods revealed that there was a strong correlation between local cases and imported cases, with *R*^2^ values above 0.8.

**Figure 6:**
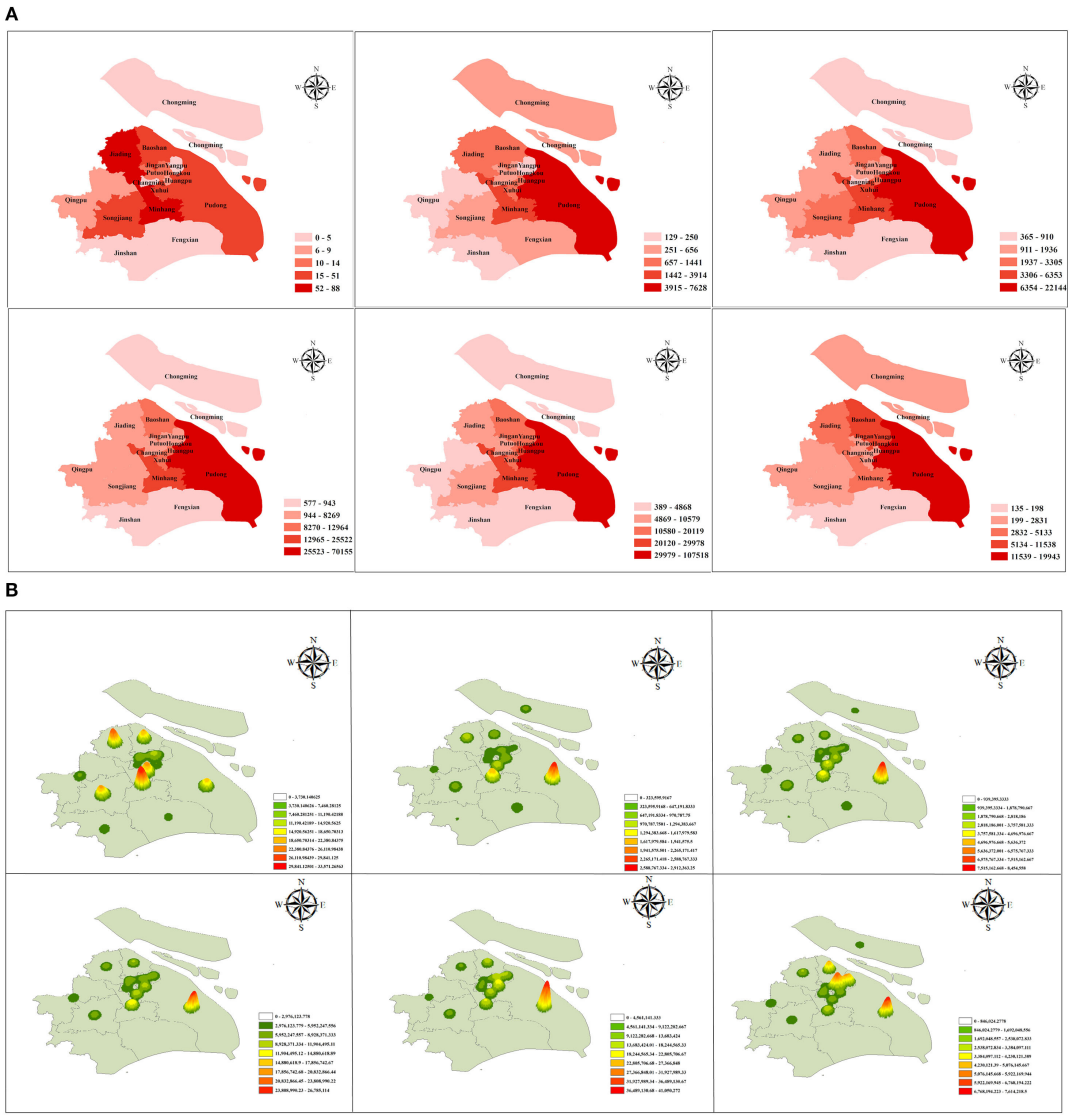
The spatial distribution of the epidemic. **(A)** A geographical map and **(B)** a nuclear density map showing the spatial distribution of local cases in Shanghai, divided into 16 districts. The six submaps represent the six stages of epidemic development. Limited by media reports, this article covers only the period from March 5 to May 31.

The spatial distribution of local cases (including local confirmed and asymptomatic infections) in Shanghai is shown in Figure 6. In the first stage, the epidemic peak occurred in the Minhang District, the Jiading District, etc. From the second stage on, the epidemic peak gradually spread to the Pudong New Area, reaching a peak in the fifth stage and decreasing in the sixth stage. Therefore, there were case reports in 16 districts of Shanghai, but there were significant geographical differences in the distribution of confirmed cases. The highest incidence rate was mainly in the Pudong New Area, followed by the Minhang District.

### Conversion analysis of asymptomatic infections to confirmed cases

**Figure 7:**
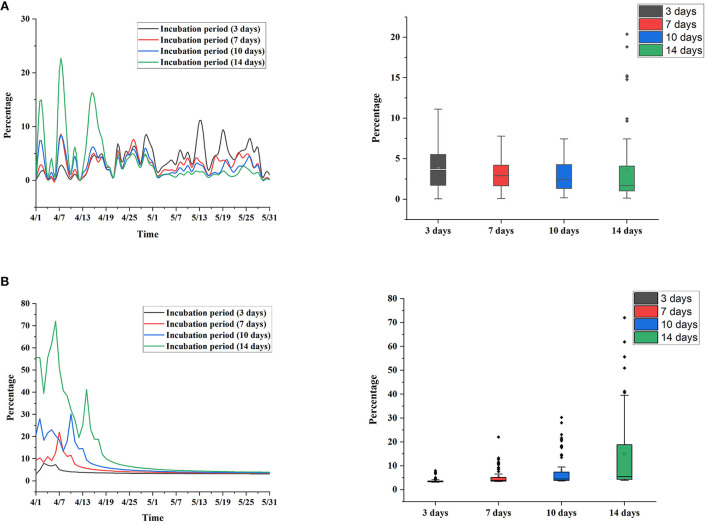
Conversion rate during each incubation period. **(A)** Actual situation. **(B)** Simulation without public health interventions.

At present, the accurate incubation period from asymptomatic infections to confirmed cases has not been determined. Therefore, we adopted a variety of settings, such as 3, 7, 10, and 14 days, to better analyze prognostic rules. In practice, because no asymptomatic infections were converted into confirmed cases in the first and second stages, the conversion rates for each incubation period for the third to sixth stages were calculated, as shown in Table 2 and Figure 7A.

**Table 2:** Conversion rate (%) during each incubation period.

	**3 days**	**7 days**	**10 days**	**14 days**
Third stage	0.74 (0.04, 1.71)	1.14 (0.09, 2.77)	2.76 (0.18, 7.43)	5.90 (0.46, 14.78)
Fourth stage	1.09 (0.10, 2.50)	2.89 (0.14, 7.79)	3.53 (0.30, 7.42)	7.99 (0.41, 20.38)
Fifth stage	3.68 (0.45, 6.83)	3.50 (0.58, 7.64)	3.56 (0.64, 5.99)	5.95 (0.70, 15.24)
Sixth stage	4.82 (0.89, 11.11)	2.93 (0.29, 5.41)	2.26 (0.17, 6.00)	1.49 (0.13, 4.94)
Total	3.80 (0.04, 11.11)	2.91 (0.09, 7.79)	2.74 (0.17, 7.43)	3.62 (0.13, 20.38)

When the incubation period was set to 3 and 7 days, the overall conversion rate of each period showed a stable trend, which was controlled between 10 and 20%. When the incubation period was set to 10 and 14 days, the change in the conversion rate in each period was more obvious, the conversion rate in the fifth stage and before was higher, and the conversion rate in the sixth stage was lower. The box chart shows the distribution of the recovery rate at each incubation period setting, and the results show that the distribution of the recovery rate was close at each incubation period setting.

In addition, we simulated the change in the conversion rate under the condition of an uncontrolled epidemic, as shown in Figure 7B. Assuming that Shanghai had not taken public health intervention measures after March 28, the case growth thereafter was calculated based on the *R*_0_ on March 28 and simulated based on a Poisson distribution. The results show that the recovery rate was higher when the incubation period was set to 10 and 14 days and gradually leveled off after May. It is speculated that public health intervention measures need ~10 days to be effective. This simulation study also verifies the importance of public health interventions.

## Discussion

### Principal results

We investigated and quantified changes in the epidemiological characteristics of COVID-19 in Shanghai as well as the effect of public health interventions. To divide the development stages for subsequent research, we used new feature vectors and dynamic time-series maps. We created a feature vector using the four indicators of local confirmed cases, local asymptomatic infections, imported confirmed cases, and imported asymptomatic infections and then drew a dynamic time-series map of interventions using the cluster analysis results and public health intervention measures. On this basis, we divided the epidemic into six stages and then dynamically and intuitively assessed the impact of public health interventions on the development of the epidemic. Compared with a subjective division (10), the stage division in this paper was scientific and objective. The subsequent *R*_*t*_ analysis also verified this conclusion. We then built a comprehensive assessment system for the process, transmission, and the impact of control measures in the analysis of COVID-19 transmission, correlation and conversion. The following points of innovation are described below.

First, we used *R*_*t*_ to perform transmission analysis and to review common public health interventions, with the goal of assessing the impact of public health interventions on the epidemic from an epidemiological standpoint. The first and second stages were defined as the “rapid rise period.” Shanghai had not implemented targeted intervention measures for cases introduced from other countries. Mobility restrictions in some key areas were implemented only in the second stage, resulting in a rapid increase in the *R*_*t*_ value. The third and fourth stages were defined as the “preliminary prevention and control period.” To control the spread of the disease and to promote the recovery of infected people, Shanghai implemented strong intervention measures, such as mobility restrictions in the city, the use of Fangcang shelter hospitals, and attention to elderly individuals, resulting in an increase in the *R*_*t*_ value. It gradually decreased after reaching its peak. The fifth and sixth stages were defined as the “control and remission period.” Shanghai also implemented measures such as conducting zoning management, optimizing the efficiency of Fangcang shelter hospitals, and standardizing nucleic acid testing on the basis of the previous measures so that the *R*_*t*_ value continued to fall. The overall level of *R*_*t*_ in the first three stages was higher than the epidemic threshold (i.e., 1) but decreased rapidly after the implementation of strict public health intervention measures. Additionally, the overall level in the last three stages was lower than the epidemic threshold. Both the decrease in the *R*_*t*_ value and the gradual decrease in the number of new confirmed cases per day indicate that the Shanghai government's interventions had a positive impact on controlling the COVID-19 epidemic and were able to effectively block the spread of the epidemic and ease the disease burden. The simulation analysis results indicate that the implementation time of public health interventions is more important, and the longer the delay is, the longer the epidemic is expected to last.

Second, for the first time, we used temporal correlation analysis to reveal the relationship between local and imported cases, with the goal of determining whether the outbreak was caused by cases imported from other countries. The spatiotemporal distribution analysis revealed the characteristics of a bimodal epidemic of local and imported cases, with local cases emerging concurrently with the appearance of imported cases. The curves fitted by various methods in the correlation analysis revealed that there was a strong correlation between local and imported cases, and the *R*^2^ value reached more than 0.8. According to the time lag analysis, there was some lag and amplification in local cases compared to imported cases, with a lag time of ~31 days. The epidemic in Shanghai was characterized by a combination of local transmission and imported cases. Therefore, it is speculated that this round of the epidemic came from imported cases. Analysis of the main causes of local infections revealed the following. (1) The spread of the epidemic was hidden and delayed. Insufficient attention was given to cryptic transmission in the early stage of imported cases. (2) The virus variant of this outbreak in Shanghai was the Omicron BA.2 mutant, which has a faster transmission speed and stronger transmission strength and can better break through the immune barrier conferred by vaccines (24–26).

Next, for the first time, we investigated how asymptomatic infections became confirmed cases. A significant feature of this round of the Shanghai epidemic was the high proportion of asymptomatic infections. The conversion of asymptomatic infections to confirmed cases is conducive to directly reflecting the impact of interventions, but previous studies on similar topics have focused less on asymptomatic infections (27–30). Therefore, our research evaluated asymptomatic patients and analyzed the relationship between asymptomatic infections and confirmed cases. When the incubation period was set to be short, the overall conversion rate of each period showed a stable trend. When the incubation period was set to be long, the volatility of the conversion rate in each period was more obvious. The conversion rate in the fifth stage and before was higher, while the conversion rate in the sixth stage was lower. The distribution of the conversion rate was similar with different incubation period settings. There are two possible causes. (1) The first is the role of public health interventions: When the incubation period is short, intervention measures may not yet come into effect. When the incubation period is long, intervention measures take effect, and then, the conversion rate gradually decreases. (2) The second is the constancy of the true conversion rate: As of May 31, the true conversion rate was ~10%. The conversion rate calculated at the setting of each incubation period was close to the true conversion rate. Furthermore, we conducted a simulation study on the change in the conversion rate under the assumption that the epidemic was not under control, i.e., the calculation was based on the R_0_ value without public health intervention measures. Additionally, the simulation study was carried out using the Poisson distribution. The effect of public health interventions was verified.

Finally, we summarized and compared our findings with those of previous studies. The analysis of transmission, correlation and conversion presented above allowed us to confirm the role of public health interventions in COVID-19 prevention and control from a variety of perspectives. To better validate the findings of this paper, we compared them to previous studies on the impact of public health interventions on the epidemic (10, 31–34). The Shanghai epidemic differed from previous epidemics in terms of viral types, the proportion of asymptomatic infections, and the intervention times. In terms of viral strains, the Shanghai epidemic was primarily caused by the Omicron BA.2 strain, which has high infectivity and rapid transmission. In terms of the proportion of asymptomatic infections, the Shanghai epidemic had a relatively high proportion of asymptomatic infections, and there was a certain proportion of confirmed cases. In terms of the intervention times, the Wuhan epidemic was controlled in 76 days, the Sichuan epidemic in 42 days, and the Shanghai epidemic in 66 days. Despite the many differences in these outbreaks, studies have shown the role of public health interventions in COVID-19 prevention and control, demonstrating their effectiveness and generalizability. Our experience can help China and other countries and regions address the prevention and control of similar infectious diseases.

### Limitations

This study still has some limitations. First, this paper describes the result of the joint action of multiple interventions. The effectiveness of a single measure cannot be assessed due to ethical requirements. Second, the corresponding clinical characteristics of confirmed cases could not be obtained. More baseline data will be collected in the future to carry out an analysis of population characteristics. Third, for other countries and regions, the outbreak and development stages of the epidemic do not necessarily show the same dynamic trajectory, and more regional and temporal analyses are needed to verify the robustness of the results.

## Conclusion

In the foreseeable future, the epidemic process will still depend on the efficiency of the implementation of public health interventions. Timely and effective public health interventions can effectively and quickly curb the spread of an epidemic and protect the health care system from the overwhelming pressure caused by the epidemic. This study describes an effective Chinese experience and can be a positive guideline for global epidemic prevention and control.

## Data availability statement

The datasets presented in this study can be found in online repositories. The names of the repository/repositories and accession number(s) can be found below: https://github.com/STUDWHJ/Shanghai.

## Author contributions

DaY: conceptualization, data curation, resources, methodology, software, formal analysis, validation, investigation, and writing—original draft. XC: methodology, software, formal analysis, validation, investigation, writing—original draft, and editing and polishing. HW: data curation, resources, software, and formal analysis. QS: software, validation, investigation, and data curation. LZ and WY: validation, investigation, and writing—polishing. PL: software, validation, and investigation. JC: investigation, resources, and writing—polishing. FL: validation, resources, and formal analysis. DoY: conceptualization, investigation, validation, resources, and methodology. YW: funding acquisition, conceptualization, investigation, resources, methodology, software, writing—review and editing, project administration, and supervision. All authors contributed to the article and approved the submitted version.
